# Socio-Technical Analysis of the Benefits and Barriers to Using a Digital Representation of the Global Horse Population in Equine Veterinary Medicine

**DOI:** 10.3390/ani13223557

**Published:** 2023-11-17

**Authors:** Tomas Rudolf Sterkenburgh, Javier Villalba-Diez, Joaquín Ordieres-Meré

**Affiliations:** 1 DEGIN Doctorate Program, Universidad Politécnica de Madrid, 28006 Madrid, Spain; 2 Independent Consultant in Veterinary Medicine, 46535 Dinslaken, Germany; 3 Faculty of Economics, Heilbronn University of Applied Sciences, 74081 Heilbronn, Germany; javier.villalba-diez@hs-heilbronn.de; 4 Department of Industrial Management, Universidad Politécnica de Madrid, 28006 Madrid, Spain; j.ordieres@upm.es

**Keywords:** digital health data, horse population, Internet of Medical Things, digital patient file, socio-technical matrix, socio-technical system analysis, veterinarian and horse owner

## Abstract

**Simple Summary:**

Technological and social progress are often closely linked. There is a consensus that future medicine will benefit from the use of available health data. The form in which these health data will be available is crucial. For a comprehensive analysis, it is necessary to harmonize and link these data and enable seamless use across system boundaries. In this paper, we consider the field of equine veterinary health data. We propose a vision that data from the entire global horse population are utilized for the benefit of animal health and longevity. With this in mind, we examine social aspects influencing technical progress. Here, we use a socio-technical matrix as a tool. We reduce the overall complexity by limiting ourselves to the following: Technically, we consider the treasure trove of data from veterinary diagnostics and the Internet of Medical Things (IoMT). Regarding social interactions, we focus on veterinarians and horse owners. Utilizing this socio-technical matrix, we branch out to identify barriers and enablers on the way to the vision. Additional elements, such as the slowly maturing awareness of horse owners regarding the value of these data as well as training of all parties in the handling of data, are identified to be crucial.

**Abstract:**

There is a consensus that future medicine will benefit from a comprehensive analysis of harmonized, interconnected, and interoperable health data. These data can originate from a variety of sources. In particular, data from veterinary diagnostics and the monitoring of health-related life parameters using the Internet of Medical Things are considered here. To foster the usage of collected data in this way, not only do technical aspects need to be addressed but so do organizational ones, and to this end, a socio-technical matrix is first presented that complements the literature. It is used in an exemplary analysis of the system. Such a socio-technical matrix is an interesting tool for analyzing the process of data sharing between actors in the system dependent on their social relations. With the help of such a socio-technical tool and using equine veterinary medicine as an example, the social system of veterinarians and owners as actors is explored in terms of barriers and enablers of an effective digital representation of the global equine population.

## 1. Introduction

The availability of health-related data in veterinary medicine for horses is on the rise due to technological advancements [1,2]. This growth mirrors the trends in human medicine [3,4]. The Internet of (Medical) Things, cloud services, and interconnected systems [2,5] are driving improvements in health monitoring and care [6]. We use the horse as a model system to illustrate our point, considering it as a representative domestic animal [7] of significant relevance and value [8]. On the one hand, it is a widespread domestic animal, with approximately 1.25 million privately owned horses in Germany as of 2019 [9]. On the other hand, horses often hold both high emotional and substantial monetary value, sometimes reaching millions of euros [10]. In the realm of veterinary medicine, a diverse range of data are gathered at various levels of aggregation during diagnostics and post-diagnosis treatment. This spans from cell- and tissue-related information (bioinformatics), and even organ- and body-part-related data (e.g., imaging informatics), all the way to data specific to individual horses (clinical informatics), and even encompasses population-related data (population informatics) [1].

Razdan and Sharma [11] defined the Internet of Medical Things (IoMT) as a network of decentralized patient monitoring devices, sensors, actuators, medical records, pharmacy controls, nutrition regimen generators, etc. These are connected to either the patient or the patient’s immediate environment and linked to a decentralized IT system, which, in turn, connects to a central IT system (e.g., a network of cloud servers and distributed storage systems). This central IT system is utilized for data collection and evaluation, and condensed information is presented to the user through a user-friendly interface (e.g., smartphone, computer). In the context of horses, the Internet of Medical Things (IoMT) is emerging as a significant source of health-related data. Examples include implanted transponder (RFID) chips for unique animal identification [12], and devices that monitor vital health parameters, like heart-rate and regularity, respiratory rate, and body temperature, offering basic insights into the animal’s well-being [13,14,15,16,17]. Additionally, there are systems for recording body movement patterns, crucial for the early detection of serious illnesses, such as infections or colic [18], as well as tools for intelligent stall interaction and (partially) automated animal husbandry, along with monitoring of feed and liquid intake [19,20,21].

The digital patient record and the equine passport provide the veterinarian with comprehensive information about the patient’s history [6], including preventive measures such as vaccinations, dental care, parasite control, or blood tests [22]. Furthermore, simulative analyses enable comparison with actual patient data, aiding lameness assessment and localization [23,24], for example. IoMT devices have also been applied to the optimization of athletic training [13] and monitoring essential indicators, like thump counts for assessing food intake and mastication [25]. While only a small percentage of animals are currently monitored partially and temporarily, the range of available monitoring systems is steadily increasing. This means there is a diverse array of equine health data accessible from both veterinary practice and IoMT devices. The technical possibilities for gathering health data are already extensive, and the following examples should, thus, be seen as a selection: gaiters with accelerometers and GPS for refining athletic training [26], a variety of heart-rate sensors for optimizing daily training [27], body temperature add-ons to identification chips [28], and comprehensive monitoring of heart-rate, body temperature, and respiration [29]. Additionally, there are solutions for lameness detection [30], activity and behavior monitoring, and thumb counting (originally developed for dairy cows) [31]. Also, sensor technology initially designed for human wearables, medical devices, and IoMT solutions is now being applied to horses for health issue detection and monitoring [32]. Furthermore, there are solutions for monitoring horse jumps for training purposes [33], heart-rate [34], and activity and performance tracking [34]. There are also options for monitoring temperature and humidity [35]. Next, we will summarize the general benefits of health-related data and explore how these benefits can be optimized. We start by examining the findings from human medicine and comparing them to the veterinary context in horses.

Much like in human medicine, health-related data hold immense potential value, but there are challenges in effective utilization. There are, however, successful approaches that offer a vision of maximizing benefits for the horse’s health, well-being, and longevity. A thorough assessment of health-related data has the potential for significant advancements in equine health [1], similar to what is apparent in human medicine [4,36]: This includes new insights for research and medical practice, more precise diagnostics, improved diagnoses, enhanced treatment decisions, improved patient care resulting in individual benefits for the patient, helping to combat diseases more effectively, developing tailored treatments, deeper understanding of illnesses, and early warnings about epidemic outbreaks. Additionally, it can lead to reductions in healthcare spending by tapping into the underutilized potential of data.

We identified approaches to collecting health-related data and harnessing the benefits of these data for the horse population. The following list is not exhaustive: The Federation Equestre Internationale (FEI) monitors horse health at FEI events [37], and in Switzerland surveillance of infectious diseases is implemented with the Equinella project [38]. Additionally, vaccination status and international vaccination compliance are tracked [39], and the Equine Infectious Disease Surveillance (EIDS) programme oversees infectious diseases in the UK [40]. The Universal Equine Life Number (UELN) identification is recorded by the World Breeding Federation for Sport Horses (WBFSH) [41], and the Equine Injury Database serves as a register of injuries on racing horses [42].

**Figure 1:**
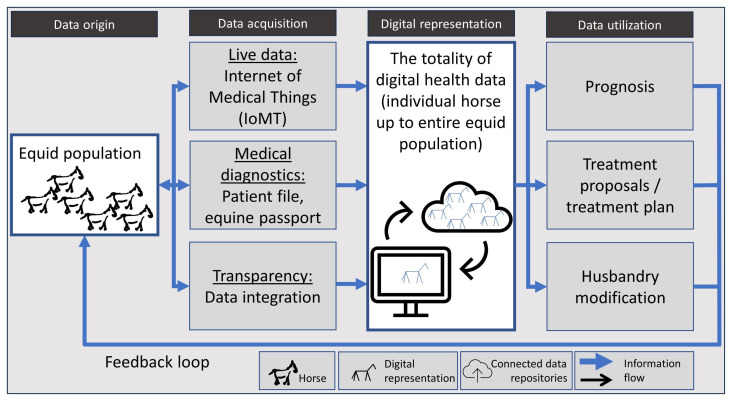
The concept of a digital representation of the health status of the global equid population.

With the aggregation of equine health data, a digital representation of the horse is, thus, increasingly taking shape as depicted in Figure 1. While we term it a “digital representation” in this paper, the entirety of data possess attributes akin to a “digital shadow” and, with established feedback, also exhibit the basic features of a “digital twin” [43,44].

We regard usable data as a valuable asset [45], and data collection can be aligned with various interests at play. The two main intentions of stakeholders in equine health data are a commercial targeting system and a non-profit commitment to animal welfare. Commercial data collection, such as from private hospitals, typically follows the goal of economic value creation (economic principle/commercial target system). For health insurance companies, a driver may be cost transparency or cost reduction (commercial target system) [46]. Corporations, whether directly linked to horses or not, may recognize the value of data as an opportunity and invest in utilization. Non-profit organizations (NPOs) are likely to prioritize the well-being of the animal (non-profit target system) [47]. The government could also contribute to data collection and utilization if there is the political will, as is already the case in human medicine (non-profit target system). This political will would need to be garnered by the group of horse owners. With both a commercial and a non-profit target system, two distinct approaches emerge for data utilization: while commercial use inherently aims for economic advantage (economic principle), non-profit use aligns with higher-level societal goals. In the context of veterinary medicine, animal welfare and longevity are two significant objectives here.

However, there is a noticeable gap in the evaluation of data that arises during collection and handling. Data collection tends to be localized, often being limited to the individual horse, local veterinary care, or to the owner receiving services from the IoMT provider. The obtained data are typically stored in widely dispersed data repositories, often in various formats with different data owners. In both human and veterinary medicine, there are four key challenges impeding consistent and seamless data evaluation [4,48]: 1. The heterogeneity of the data: This necessitates additional processing steps before a comparative analysis. 2. The distributed storage: Data are stored in different repositories, including cloud storage, servers in veterinary practices or clinics, and even private storage locations of owners and breeders. 3. A need for collaborative communication: There is a requirement for more open and cooperative communication among various stakeholders. 4. Data quality and interoperability: Ensuring data quality and compatibility across different systems is crucial.

Similar to human medicine, the data available can, thus, be described as “not sufficiently utilized” in terms of their potential benefits for equine health. This limitation hinders the optimal use of information for the animal’s welfare and health maintenance [49].

From the gaps identified above, we derive the following two research questions:

RQ1: Is there a feasible approach to enable the full potential of the data in this domain? and,

RQ2: Is there an option to apply an organizational approach to investigate barriers and enablers in achieving the vision?

The subsequent sections of the paper will delve into the adopted methodology (Section 2), and Section 3 will introduce a framework for the analysis of the socio-technical system of health-related equine data. Beginning with a technical scale, we integrate a social scale drawn from the literature to form a socio-technical matrix. This matrix underwent reviews from veterinarians and horse owners as key actors in the system. The insights gained from these reviews were then integrated into the overarching concept. In Section 4, we present an analysis of the socio-technical system, and highlight findings regarding barriers and enablers in achieving the vision. Finally, in Section 5, we draw conclusions and provide suggestions for future directions.

## 2. Methodology

As presented in [50], human needs and preferences play a pivotal role in influencing technological advancements, which, in a reciprocal manner, have significant effects on human society. The existing literature has identified three distinct modes in which social values interplay with technology, namely embodied, exogenous, and interactional.

In addition to these modes, historically, several design methodologies have been employed in the incorporation of social, ethical, and cultural principles into information system design. The focus is to analyze the ways these approaches enrich our comprehension of infusing human values and frame the context for the proposed research. All of these methodologies are similar in the way that they focus on designing the system based on some ethical, cultural, or social values, like value-sensitive design [51,52].

The value-sensitive design methodology maintains continuous oversight of the design process by incorporating values into the design through conceptual, technical, and empirical investigations. Conceptual inquiries provide insights into the competing values that should be incorporated into the system design, such as the balance between anonymity and trust. Technical examinations explore how technology facilitates specific human activities and values. Empirical investigations involve observing users’ actions. This approach does not prioritize user requirements in the process; instead, it assesses the potential impact of these design choices on users. Critics of this method frequently argue that it promotes the perspective that values are technology-centered, meaning that technology supports certain values while undermining others. They also contend that values do not merely flow through society and become integrated into systems; rather, the users and society influence technological development, just as technological developments influence them. Furthermore, it is often criticized for placing excessive emphasis on the usability of the final system.

We have found this approach suitable for constructing the framework we think is needed in order to address the research questions adopted earlier, in particular the conceptual dimension involved, with enough focus on both the technical and social dimensions.

The concept of a socio-technical system, originally developed by Trist and Bamforth [53], has found applications in a diverse range of fields, including occupational safety [54,55,56], Industry 4.0 [57,58,59], organization development [60,61], and psychology [61,62]. Lean management systems as defined by Shah and Ward [63] also drew on the socio-technical approach. Kaldor’s original introduction of mapping technical progress to a scale is widely adopted [64], and Covey [65] further defined a social scale as a sequential cascade toward organizational alignment. Schmidt and Villalba-Diez connected both axes to a matrix. Schmidt incorporated a third axis of digitization [66,67]. For our investigation into the socio-technical system encompassing owners and veterinarians as social actors, along with the digital representation of the horse population as a technical object of study, we employed these social and technical scales. We constructed a socio-technical matrix, introducing a business-oriented tool for socio-technical analysis [68].

This matrix allows us to dissect barriers and enablers in realizing the vision of a digitally represented global equine population, taking into account Trist and Bamforth’s hypothesis that progress toward this vision hinges on the interplay of social and technological advancement.

This concept facilitates the vision of a digital representation of the global equine population. Indeed, it promotes the consideration of such digital representation as a system of interconnected, harmonized data instances where all available data on the global horse population involved are linked through ontologies [69], with the aim of enabling the full potential of the data in the domain.

## 3. Socio-Technical Framework for the Analysis of Horse Data Utilization

A technical scale and a social scale were introduced as an enhancement of the literature, and a combination of both into a socio-technical matrix was proposed as a tool for further analysis of the socio-technical system. The technical scale described three levels of aggregation of health data from the individual horse to the global entirety of horses. The social scale leads from individual characteristics to interpersonal networks to personal advancement and the alignment of actors toward common goals. By combining the two scales into a matrix, technical and social progress were analyzed in their interrelations according to selected themes.

In the following, the people involved in the socio-technical system are referred to as “actors”. The horse owner and the veterinarian are singled out as important actors in this context, since both are in direct contact with the horse and take responsibility for the welfare and longevity of the animal. Both actors are the subject of this study. Other actors and groups of actors are deliberately not or are only marginally considered for reasons of focus and reduction in complexity. These include health insurance companies, veterinary practices, providers of practice software, the pharmaceutical industry, IoMT service providers, and research institutions. The next Section presents a four-step social scale based on the literature.

### 3.1. The Social Scale

**Figure 2:**
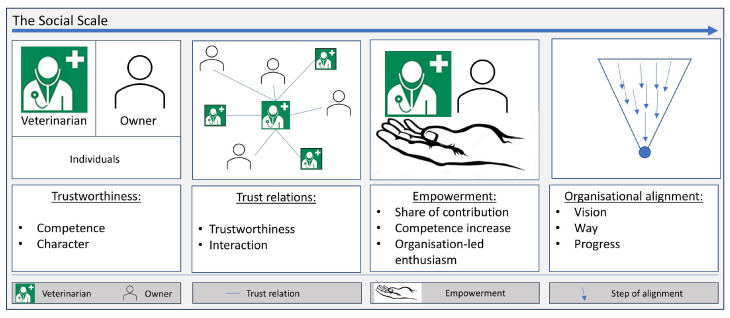
The social scale, ranging from trustworthiness to trust relations and empowerment to organizational alignment.

The social scale considers the social aspect of the interaction of individuals, beginning with individual prerequisites, through interpersonal relations and the necessary personal development, to the consolidation of common interests. Covey [65] argues that trustworthiness, based on competence and character, is a prerequisite for trust relations. He describes the establishment of trust relations as a prerequisite for empowerment, which, in turn, is a prerequisite for alignment. These four items, i.e., trustworthiness, trust relations, empowerment, and alignment, make up a social scale, as presented in Figure 2. At the first level of this social scale, Covey placed the trustworthiness of actors as a prerequisite for social progress. Trustworthiness is broken down here in terms of the system under consideration and in terms of equine health data. This designates the basic prerequisite for actors to be involved in the system in terms of the social fabric. Detached from horse health data, a basic requirement for perceived trustworthiness is translated into the quality that thinking, speaking, and acting are in harmony. With respect to equine health-related data, we interpret trustworthiness first as a willingness to commit in this harmony and that the data will be used in the best interests of equine health and welfare. This should be carried out in a competent manner so that the expected benefits are achieved. Misuse of data must be prevented accordingly. For the veterinarian, trustworthiness rests on three additional pillars: (i) A personal value system that reflects societal norms. (ii) Professional ethics in the sense of the Hippocratic Oath [70] or the Geneva Pledge [71]. (iii) Professional competence.

The next section describes how trustworthiness enters into trust relations. Trust relations describe relationships between actors that are established and confirmed on the basis of trustworthiness. The trust relations between veterinarians and horse owners as representatives of the patient and with their networks are key. Trustworthiness at the first level of the social scale forms the basis for the emergence of trust relations. When trustworthiness is confirmed bilaterally by the owner and the veterinarian, trust relations grow, and purposeful cooperation can take place. Mutual access to data can be granted on the basis of established trust relations for the benefit of the horse. Both owner and veterinarian are, in turn, embedded in social structures. The veterinarian’s social system also includes their own organization with staff and colleagues. In addition, most veterinarians have social relations with colleagues and specialists outside their own organization. Animal owners are also networked. Stable communities, sports clubs, and interest groups are examples of this. These relationships form a larger network of trust. The following section explains empowerment in this context.

The third level on the scale is empowerment. Empowerment describes the development of stakeholders to enable them to contribute increasingly to the achievement of the vision. Covey interpreted empowerment as giving free rein to an actor’s self-determined motivation to contribute to the achievement of a vision with self-control, self-management, and self-organization. He equates empowerment with organization-led enthusiasm. The vision has already been described as the state in which the health data of the entire equine population are best utilized to achieve longevity and health maintenance. The prerequisites for this were identified as connectedness, harmonization, and the interoperability of data and data repositories. Empowerment thus describes the attainment of competence and confident data management and evaluation. Inspiration and motivation are key to both. This leads to the final level of the scale, which is dedicated to alignment.

The fourth and final level of the social scale is devoted to “organizational alignment”. Alignment is a process that describes the improvement of orientation towards a common goal. In the given context, alignment describes the increasing orientation of a group of actors towards the vision. Animal welfare, optimal use of data, and protection of personal rights, but also commercial benefits can be possible objectives. Alignment is achieved when actors in a system, or a subsystem to a larger system, develop a common understanding of an issue through empowerment and alignment on goals and methods. Alignment is not a one-time process, but an ongoing effort that interacts with empowerment. External influences and developments may lead to adjustments to the direction of the goal or changes in the strategy for achieving the goal. The representation of the social scale is shown in Figure 2.

Next, the technical scale is presented. The technical scale deals with the digital representation of horses and leads from the representation of the individual horse to groups of horses with already linked health data up to a vision of the digital, harmonized, networked, and interoperable representation of the global horse population.

### 3.2. The Technical Scale

**Figure 3:**
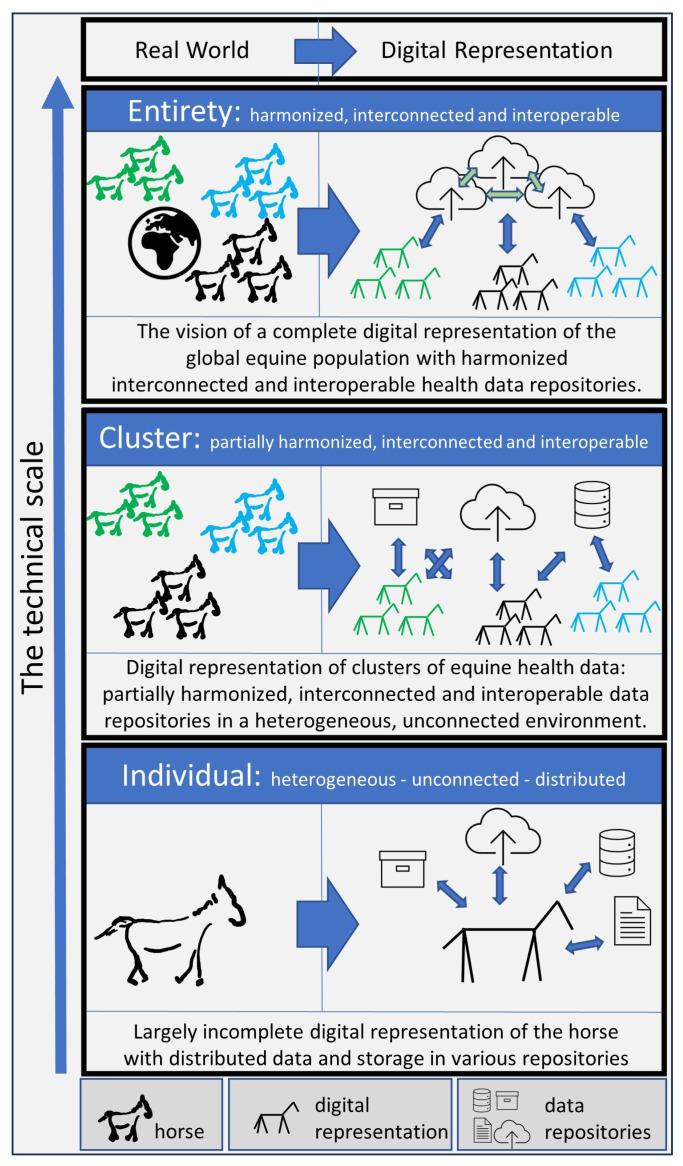
Technical scale, starting at the bottom with the digital representation of the individual horse and ending at the top with the vision of a globally connected and harmonized digital representation of the entire equine population. Harmonized, merged, and connected clusters are shown at the second level.

The first level of the technical scale is the individual horse health data system. Here, the described technical possibilities for recording individual horse health data are used as they are already applied in many cases. The data construct referred to as the digital representation of the individual horse thus represents the first level of our technical scale. The top level of the technical scale describes the sum of all potentially collectible health-related data for the entire global equine population in a usable and exploitable form. It outlines the vision of an ideal state with achieved harmonization, interconnectedness, and interoperability of data. In between is an intermediate level with clusters of harmonization, networking, and interoperability, representing lighthouse projects which have already been successfully used in the past to investigate or analyze specific clinical characteristics based on previously collected data. In this context, the initiators have succeeded in overcoming the challenges described, at least with regard to the underlying object of investigation, and in combining the data in a way that makes evaluation possible on an as-needed basis. The complete scale is shown in Figure 3.

Both scales are now related and combined into a matrix.

### 3.3. The Socio-Technical Matrix

**Figure 4:**
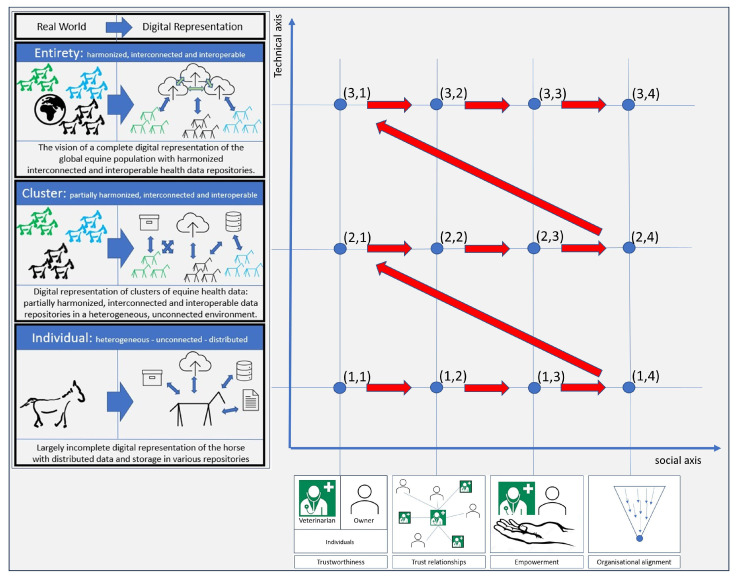
The technical scale is the transformation of the real world into a digital representation and the combination with the social scale to form a socio-technical matrix. The blue intersections represent potential fields of action. The red path describes the way to the vision.

Crossing both scales as proposed by Schmidt [67] and Villalba-Diez [66] leads to the socio-technical matrix representation (Figure 4) as it has been used to systematically explore barriers and enablers. Intersections in the matrix describe potential fields of action, where a technical characteristic meets a social component. The analysis of selected intersections revealed barriers and enablers. Trist and Bamforth [53] already stated the need for social progress to align with technical progress. By passing through the four points of the social scale before taking the next step on the technical scale, a sustainable path through the matrix can be taken because of its social underpinning. This results in the red path through the matrix shown in Figure 4.

In the following, different development possibilities of the overall system are traced by means of runs through the matrix. Technical level 1 is analyzed on the basis of the social progress of individual actors. At the second technical level, groups of actors are examined, while at the third technical level, the entirety of global actors are considered. Statements are based on information gathered from informal discussions held with seven veterinarians and six horse owners.

In Section 4, the concept of socio-technical systems analysis is applied to the system of equine health-related data. The proposed path through the matrix towards the vision is taken, and barriers and enablers are analyzed. Alternative paths are highlighted in terms of the achievability of the vision.

## 4. Results and Discussion

As we move through the matrix, barriers and enablers within the socio-technical system on the path to achieving the vision are highlighted below, along with key influencing factors. Social and technical aspects were examined. To analyze the socio-technical system, we first followed the proposed approach presented in Figure 4 and highlighted selected elements. These paths can help the actors not just to identify barriers and enablers but also to define their own strategy according to their own willingness within a standard and predictable context. Alternative routes were then considered.

### 4.1. Transition from Trustworthiness to Trust Relations on the Individual Level

The trustworthiness of the horse owner and the veterinarian is the starting point of the considerations. On the individual level of the technical scale, the trustworthiness of the individual actors was identified as a prerequisite for trust relations. In the given context, trustworthiness is an initially inherent property of the actors, which is not directly visible to the outside world and, therefore, has to be confirmed or proven iteratively in the process of establishing a trust relation. This complex process of establishing trust relations and confirming trustworthiness can be accelerated externally: (i) The reputation of a veterinarian as an indication of future behavior represents an externally perceptible clue [72]. (ii) Mutual expectations can be reconciled through contractual agreements to align the scope of services and other expectations [73]. Reputation was cited as a dominant factor in the initial assessment of veterinarians’ trustworthiness. Over the course of the relationship, trust grows as long as the trustworthiness of the actors is confirmed. Positive experiences confirm the presence of trustworthiness and, thus, promote the development of trust relations. Conversely, negative experiences can damage or destroy trust relationships. These mainly originate from the areas of professional competence, character traits, or commercial processing. In addition, the veterinarian is expected to confidently assess the limits of their own abilities. Mutually perceived trustworthiness among horse owners often involves a leap of faith in the sense of a positive attitude: As trust relations are subsequently established, trustworthiness is examined and evaluated in more detail. Differences in personal value systems and behaviors appear to be the greatest barriers to establishing trust relations between owners. The principle of trust relations is supported by the resulting reduction in complexity in a complex environment characterized by uncertainty. Focusing on the individual horse’s health data, the veterinarian is expected to document the examination results conscientiously and completely, while the owner’s statements—e.g., during the anamnesis—are expected to be correct. Establishing a treatment contract, similar to a contractual relationship with IoMT providers, serves as a second path to accelerate the establishment of trust relations based on the alignment of mutual expectations. Treatment errors are a barrier that can undermine trust in the veterinarian. The industry’s concept of "error as an opportunity", has been affirmed in principle but quickly reaches its limits when it comes to one’s own horse. Even though the financial loss is usually covered by insurance, the emotional damage is not. A previously established stable trust relation based on proven trustworthiness can help in such a crisis. A facilitator for trust relations that supports the quality of the digital representation of the individual horse at levels 1 and 2 is proper documentation and data collection, as a task provided and paid for by the client, as a necessity to avoid risks for the animal, and as a reliable interface with veterinary colleagues. An example of standardization of language rules can be found in the Nomina Anatomica Veterinaria (NAV) of the World Association of Veterinary Anatomists (WAVA) [74].

### 4.2. Empowerment and Organizational Alignment on the Individual Level

Empowerment and organizational alignment are closely related, similar to trustworthiness and trust. Empowerment and alignment in the context of veterinary health data address the question of how to further empower users and veterinarians in dealing with the digital representation of the animal and how to achieve alignment in terms of contributions to achieving the vision. Alignment requires empowerment, and, at the same time, progress in alignment triggers new empowerment needs. On the part of the owner, the veterinarian in their role as a service provider is expected to take over the data collection in the context of diagnostics with the necessary professional competence. Such skills were assumed and expected to work on legal regulations that require regular training. All owners interviewed wanted to be involved in critical treatment decisions in the best possible way after detailed consultation. This consultation can be seen as case-related empowerment of the owners. A barrier to organizational alignment arose from the different assessments of data ownership of the digital representation of the individual animal; while all owners were of the opinion that they were entitled to the health data of their horses, veterinarians also claimed data ownership with regard to evaluation, e.g., for research purposes. One veterinarian pointed out that the feed industry is showing interest in veterinary organizations and that large companies are acquiring clinics and hospitals. He saw this as a potential attempt to gain access to health data. Commercial interest was suspected.

### 4.3. From Trustworthiness to Trust Relations on the Cluster Level

Trustworthiness is also a prerequisite for trust relations at the cluster level. Here, at the second level of the technical scale, trustworthiness and the ability to establish trust relations were assumed to be more complex than in the local environment of the individual horse because of the significantly larger group and because of greater distances and wider regional distribution. Clusters often have an organizational core that plays an essential role as a hub of trust. In Germany, examples include the German Equestrian Association and the Society for Veterinary Medicine, which provide some of the coordination between groups of owners or veterinarians in terms of veterinary issues and health-related data. However, the role of these organizations was considered "small" in relation to the overall task. All respondents saw room for improvement. Trust relations are always subject to personal risk mitigation, which can be a barrier.

### 4.4. Empowerment and Organizational Alignment at Cluster Level

Similar to the owner who leaves the evaluation and handling of individual health data in the hands of the veterinarian and only requires involvement for essential decisions, the ability to evaluate data pools at the cluster level was also seen more as a task for specialists. Researching veterinarians addressing a cluster-specific question were included in this group, as were university and pharmaceutical researchers. It was generally assumed that the ability to analyze mass data requires special training and that veterinarians without such skills are more likely to be involved in the investigation of case-study-type research. Thus, obtaining the necessary skills is another barrier to the use of appropriate data pools, while the benefit of such evaluations was assessed as "high" and named an enabler. Supplementing the interviews, it should be noted that health information literacy is becoming more prevalent in veterinary education and professional life and that, recently, new learning formats have been used to teach these skills [75,76]. Thus, university education also makes a significant contribution to the empowerment of (veterinary) medical students [77,78,79].

### 4.5. Trustworthiness as a Prerequisite for Trust Relations on the Global Level

While it still seemed relatively easy for the interlocutors to visualize certain clusters and the utility of evaluating cluster-specific equine health-related data, the third level of the technical scale entered a level that required explanation. Trustworthiness in a global context, i.e., an enormously large group, across cultures and countries, was seen as perhaps only mappable through organizations (e.g., NPOs). No other avenues were identified. Commercial use of the data, e.g., by the feed industry or other market players, was considered to be detrimental compared to only animal welfare use, unless this commercial use does not limit the non-commercial use. There is a potential conflict of objectives due to the additional economic objective.

It was agreed that education of owners about the value of health-related data should be an important next step towards the vision of further non-profit, welfare-oriented use. It was concluded that regulations similar to those implemented for humans regarding the protection of personal data would be desirable for horses. The European General Data Protection Regulation was cited as an example. Similarly, several discussion threads ended with the realization that political will is essential to achieve legal regulation for horses/animals. This political will, in turn, could be achieved through an appropriate level of organization of horse owners and representation of interests through organizational nodes. In addition to the interview findings, it can be stated that political will formation is a complex endeavor. Weible [80] describes it as the starting point of the policy cycle. The formation of political will involves defining the problem (problem awareness) and then addressing it. Associations, interest groups, and political parties are playing an important role in supporting this alignment and in expressing and addressing the political will.

Alternatives to the red-marked path through the matrix were explored. This process confirmed that the proposed path promises a high degree of sustainable progress since necessary social developments are completed before the next step on the technical scale. Deviations from this path are conceivable, for example, in the case of commercial data use. If data were to become available to large corporations, e.g., through the acquisition of veterinary practices and clinics, larger data repositories would come under the control of commercial actors. Data would then be available to a commercial entity for commercial use, and animal health objectives would be secondary. Steps (1.1) through (2.4) can then be followed within the commercial organization. However, achieving the vision then encounters the difficulty of consolidating repositories globally. On the other hand, health data management in the hands of NPOs is possible if funding can be secured for the necessary technology and human resources. Political will transferred into governmental support can provide the funding.

## 5. Conclusions

We applied a socio-technical system approach developed in the business world to the field of equine health data. We outlined a vision of a global, interconnected, and harmonized digital representation of the equine population. Using the socio-technical approach, we were able to propose a standardized framework enabling a sustainable, transparent, federated, and coordinated method of taking benefits from the digital equine datasets and to identify barriers and enablers on the way to achieving the vision. In this way, and according to the results and discussion section, the two identified research questions have been answered.

A sustainable approach to the development of the system, involving the social system of horse owners and veterinarians, seems to us to be more sustainable—and in the long run more successful in terms of animal welfare and longevity—than the commercial exploitation of the data. However, building political will, self-organization, and the financing of this approach is a major challenge.

The proposed framework helps to provide a context for data exchange within a consistent relational structure that ensures good governance principles and supports the convenience of data sharing. In this way, all of the participants have a clear view of the benefits and challenges the other participants have in relation to data. They can better understand the value contribution within a defined context. Thus, the framework can help the standardization of relations and data flows, ultimately increasing the value created by integrated data analyses. It also simplifies the complexity of the overall system by focusing on pairs of two selected actors.

This simplification, in turn, also leaves some aspects of the overall system that remain unexamined: political will formation could have been analyzed in more detail. For the commercial utilization in particular, incentive- or disincentive-based activation of the owners and veterinarians requires further exploration. Within the whole ecosystem, we identified about 20 actors and actor groups. Family veterinarians, veterinary experts, owners, breeders, health insurance companies, the pharmaceutical industry, IoMT providers, data analysts, clinics and hospitals, providers of practice software, the feed industry, associations, interest groups, parties, and many more make up a complex network with various value streams, money streams, knowledge and information streams, risk streams, and service streams worthy of investigation. An analysis of these aspects exceeds the scope of this paper by far. However, we see this analysis as a possible step beyond the research presented here toward greater practical relevance and more actionable results. Information was collected through semistructured interviews with veterinarians and owners. A formal and larger interview-based study with an extended group of actors would allow for additional, statistical analysis and increased relevance.

The limitation to horses seems arbitrary but is justified by the value and importance of this animal. A broader examination of livestock might have brought additional aspects to light but would have also greatly increased the complexity of the study. Therefore, further research is needed to verify the usefulness of the socio-technical matrix framework when extended to other contexts.

The study was essentially based on the situation in Europe, which means that a more global perspective could be beneficial if other scenarios can provide additional insights that can increase the robustness of the framework.

## Data Availability

The data are not publicly available due to privacy restrictions.

## Abbreviations

The following abbreviations are used in this manuscript:

IoMT	Internet of Medical Things
RFID	Radio-Frequency Identification
NPO	Non-Profit Organization
